# Quality of Information Provided by Artificial Intelligence Chatbots Surrounding the Reconstructive Surgery for Head and Neck Cancer: A Comparative Analysis Between ChatGPT4 and Claude2

**DOI:** 10.1111/coa.14261

**Published:** 2024-12-04

**Authors:** Paolo Boscolo‐Rizzo, Alberto Vito Marcuzzo, Chiara Lazzarin, Fabiola Giudici, Jerry Polesel, Marco Stellin, Andrea Pettorelli, Giacomo Spinato, Giancarlo Ottaviano, Marco Ferrari, Daniele Borsetto, Simone Zucchini, Franco Trabalzini, Egidio Sia, Nicoletta Gardenal, Roberto Baruca, Alfonso Fortunati, Luigi Angelo Vaira, Giancarlo Tirelli

**Affiliations:** ^1^ Department of Medical, Surgical and Health Sciences, Section of Otolaryngology University of Trieste Trieste Italy; ^2^ Unit of Cancer Epidemiology Centro di Riferimento Oncologico di Aviano (CRO) Istituto di Ricovero e Cura a Carattere Scientifico (IRCCS) Aviano Italy; ^3^ Unit of Otolaryngology AULSS 2‐Marca Trevigiana Treviso Italy; ^4^ Section of Otorhinolaryngology, Department of Neurosciences University of Padova Padova Italy; ^5^ Unit of Otorhinolaryngology – Head and Neck Surgery “Azienda Ospedale Università di Padova” Padova Italy; ^6^ Department of ENT Addenbrooke's Hospital, Cambridge University Hospitals NHS Foundation Trust Cambridge UK; ^7^ Department of Otorhinolaryngology Meyer Hospital, I.R.C.C.S., University of Florence Firenze Italy; ^8^ Maxillofacial Surgery Operative Unit, Department of Medicine, Surgery and Pharmacy University of Sassari Sassari Italy

**Keywords:** AI, artificial intelligence, ChatGPT4, Claude2, head and neck Cancer, reconstructive surgery

## Abstract

**Introduction:**

Artificial Intelligences (AIs) are changing the way information is accessed and consumed globally. This study aims to evaluate the information quality provided by AIs ChatGPT4 and Claude2 concerning reconstructive surgery for head and neck cancer.

**Methods:**

Thirty questions on reconstructive surgery for head and neck cancer were directed to both AIs and 16 head and neck surgeons assessed the responses using the QAMAI questionnaire. A 5‐point Likert scale was used to assess accuracy, clarity, relevance, completeness, sources, and usefulness. Questions were categorised into those suitable for patients (group 1) and those for surgeons (group 2). AI responses were compared using *t*‐Student and McNemar tests. Surgeon score agreement was measured with intraclass correlation coefficient, and readability was assessed with Flesch–Kincaid Grade Level (FKGL).

**Results:**

ChatGPT4 and Claude2 had similar overall mean scores of accuracy, clarity, relevance, completeness and usefulness, while Claude2 outperformed ChatGPT4 in sources (110.0 vs. 92.1, *p* < 0.001). Considering the group 2, Claude2 showed significantly lower accuracy and completeness scores compared to ChatGPT4 (*p* = 0.003 and *p* = 0.002, respectively). Regarding readability, ChatGPT4 presented lower complexity than Claude2 (FKGL mean score 4.57 vs. 6.05, *p* < 0.001) requiring an easy‐fairly easy English in 93% of cases.

**Conclusion:**

Our findings indicate that neither chatbot exhibits a decisive superiority in all aspects. Nonetheless, ChatGPT4 demonstrates greater accuracy and comprehensiveness for specific types of questions and the simpler language used may aid patient inquiries. However, many evaluators disagree with chatbot information, highlighting that AI systems cannot serve as a substitute for advice from medical professionals.

**Summary**

- Artificial Intelligences (AIs) are changing the way information is accessed and consumed globally.
- ChatGPT4 and Claude2 had similar overall mean scores of accuracy, clarity, relevance, completeness and usefulness, while Claude2 outperformed ChatGPT4 in sources.
- When dealing with questions that could be raised by head and neck surgeons, Claude‐2 demonstrated a significantly lower mean accuracy and completeness scores compared to ChatGPT4.
- Regarding readability, ChatGPT4 presented lower complexity than Claude2.
- Given that a significant portion of evaluators expressed disagreement with the information offered by chatbots, these AI systems require refinement and cannot serve as a substitute for advice from medical professionals.

## 1 Introduction

Head and neck cancer represents one of the major challenges for global health with the economic cost of diagnosing and treating this disease being burdensome [1]. Addressing head and neck cancer often requires a multidisciplinary approach, involving various professionals such as head and neck surgeons, radiation oncologists, and medical oncologists [2]. Since this pathology often presents at an advanced stage, surgical therapy may require extremely complex reconstructive techniques [3]. This raises profound questions for both patients who must undergo the procedure and the surgeons who perform it. On one hand, these advances allow us to push the boundaries of what is medically achievable further and further. On the other hand, they require clear and transparent communication about the risks and benefits involved [4]. As the complexity of these procedures grows and with the abundance of information online, more and more patients may be turning to digital resources to inform themselves [5].

The introduction of artificial intelligence‐powered chatbots, particularly those based on complex language models like ChatGPT (from OpenAI in San Francisco, CA, USA) and its successor Claude2, has significantly changed the way information is accessed and consumed globally since their release in late 2022. These platforms have rapidly gained popularity, with ChatGPT reaching 100 million users in just 2 months after launch [6]. By July 2023, Claude2 emerged as its competitor [7]. Processing vast datasets containing billions of data points and trillions of words, these advanced AI systems mimic human language capabilities, demonstrating their transformative impact in areas such as healthcare [8, 9, 10, 11, 12, 13, 14].

While these platforms can offer immediate and personalised information, the quality of their content in specialised medical fields has not been thoroughly evaluated. Misinformation in oncology can have serious consequences, such as replacement of the evidence‐based treatments options with alternative approaches, treatment delays or incorrect management decisions [15].

This study aims to evaluate the quality of informative content provided by ChatGPT and Claude2 in the field of reconstructive surgery for head and neck cancer, in order to identify the strengths and weaknesses of each platform.

## 2 Materials and Methods

### 2.1 Question and Answer Development

A collaborative effort by three researchers (PBR, AVM, and GT) led to the formulation of a set of 30 questions (found in Supporting Information 1 and 2) covering various aspects of reconstructive surgery related to head and neck cancer. These questions were reviewed and refined by the team to eliminate any potential inaccuracies or ambiguities, achieving consensus on their final form. The set was organised into two categories: the first group (group 1) included questions that could be posed by patients. The second group comprehended questions that could be raised by head and neck surgeons (group 2). Both ChatGPT4 and Claude2 were tasked with answering these questions, with responses being collected for further analysis. The questions in the first group were prompt engineered with the instruction, “Please provide patient education material in response to the following question, ensuring it is accessible at a 5th‐grade reading level.” For both groups of questions, a second request was made after the initial answer was given, using the phrase: “Please provide bibliographic references to your sources.” For each question posed, a fresh chat instance was generated to ensure that the responses remained uninfluenced by previous answers.

### 2.2 Evaluation Method

The evaluation involved 16 head and neck surgeons who assessed the answers using the Quality Assessment of Medical Artificial Intelligence (QAMAI) tool [16]. This was to gauge the quality of information provided by the artificial intelligence systems. The assessment covered six criteria (accuracy, clarity, relevance, completeness, sources, usefulness) with scores ranging from 1 (strongly disagree) to 5 (strongly agree). Additionally, the responses to the first set of questions were analysed using the Flesch–Kincaid Calculator to verify adherence to the 5th‐grade reading level requirement [17]. The study was performed in alignment with the Helsinki Declaration principles and did not necessitate ethics committee approval, as it involved neither patients nor animals.

### 2.3 Statistical Analysis

The evaluation by experts was detailed both collectively and individually across the six criteria, with scores categorised into three bands: disagree (including strongly disagree), neutral, and agree (including strongly agree). The comparison between ChatGPT4 and Claude2's performances considering categorical scores, was conducted using the McNemar test. For each expert, an overall score was further calculated summing up the rates over the 36 questions; overall score was summarised as mean values and differences between chatbots were compared though paired Student's *t*‐ tests. The inter‐rater reliability was assessed through the Intraclass Correlation Coefficient (ICC), treating scores as ordinal data. ICC estimates and their 95% confidence intervals were determined based on a 2‐way random‐effects model with absolute agreement from individual surgeons' ratings. ICC values were interpreted as follows: below 0.50 as poor, between 0.50 and 0.75 as moderate, between 0.75 and 0.90 as good, and above 0.90 as excellent. Paired Student's *t*‐test test was utilised to compare the performances of ChatGPT4 and Claude2 based on the Flesch–Kincaid readability scores.

We used R version 4.2.3 (R Core Team 2023) and the following R packages: Likert (version 1.3.5), irr (version 0.84.1) and DescTools (version 0.99.54). All statistical tests were two‐sided and *p* < 0.05 were considered statistically significant.

## 3 Results

Of the 20 head and neck surgeons contacted, 16 agreed to participate and evaluated all the responses (found in Supporting Information 1 and 2) in accordance with the QAMAI questionnaire. Initial comparisons between the two chatbots were made for each question by considering the cumulative scores collected for each parameter. In group 1, 10 out of 15 questions have significantly divergent scores between ChatGPT4 and Claude2, and 9 out of those 10 exhibited a higher score for Claude2. When considering group 2, only 4 out of 15 answers have significantly different scores, and furthermore, they display contrasting trends compared to group 1 (e.g., for question Q2: in group 1, the score is higher for Claude, while in group 2, it is higher for ChatGPT) (Supporting Information 3).

A further comparison among chatbots was conducted by analysing scores across different parameters (Table 1). When examining the entire set of 30 questions, discrepancies in scores between the two chatbots were evident solely for the sources metric, wherein Claude exhibited superior performance (average score 111.0 vs. 92.1; *p* < 0.001). However, upon stratifying the data by the two Groups, Group 2 exhibited statistically significant disparities in accuracy (*p* = 0.003) and completeness (*p* = 0.002) scores, with ChatGPT attaining higher values for these metrics.

**TABLE 1:** Overall evaluation (mean scores value) of ChatGPT4 and Claude2.

Parameter	Overall			Group 1			Group 2		
	ChatGPT4	Claude2	*p* *	ChatGPT4	Claude2	*p* *	ChatGPT4	Claude2	*p* *
Accuracy	113.9	110.9	*p* = 0.203	54.80	55.63	0.632	59.13	55.31	0.003
Clarity	128.9	128.1	*p* = 0.714	61.94	62.63	0.716	67.00	65.44	0.191
Relevance	120.3	120.2	*p* = 0.981	59.25	60.43	0.482	61.00	59.75	0.396
Completeness	115.0	110.0	*p* = 0.089	56.69	56.57	0.950	58.31	53.44	0.002
Sources	92.1	111.0	*p* < 0.001	43.06	58.50	< 0.001	49.00	52.50	0.046
Usefulness	116.4	115.1	*p* = 0.586	59.44	59.63	0.898	57.13	55.31	0.219

* Paired Student's *t*‐test.

**FIGURE 1:**
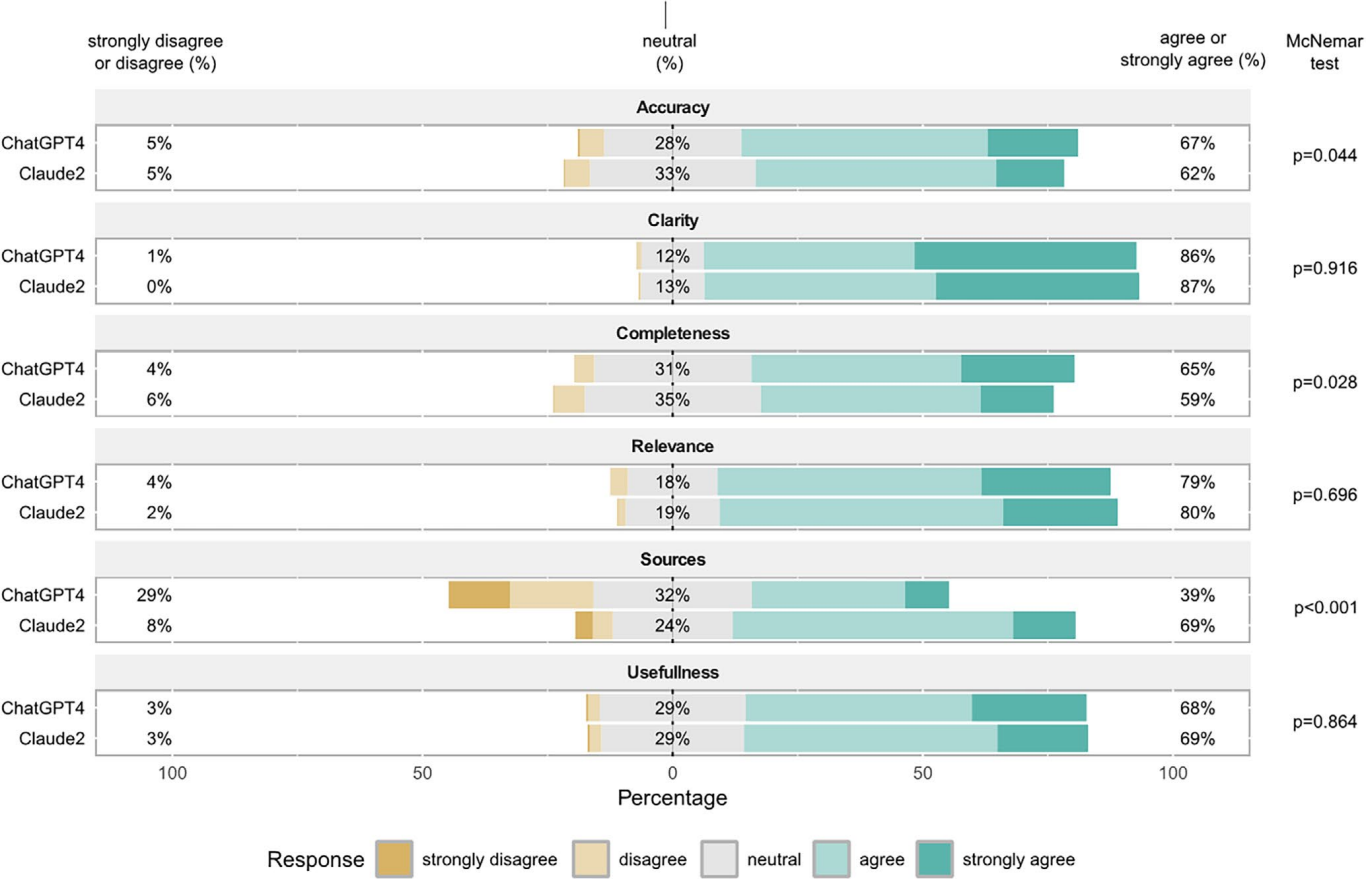
Barplot for likert items respect to Claude2 and ChatGPT4. On the left the % refer to score 1–2, in the middle to score 3 on the right to score 4–5.

We evaluated the percentage of raters reporting agreement or strong agreement (score 4–5) with responses from Claude2 and ChatGPT4 (Table 2). Overall, Claude2 presented a higher statistically significance percentage of agreement/strong agreement than ChatGPT4 (70.7% vs. 67.4%, *p* = 0.002), however when we analysed the scores according to the parameters, this result is consistent only for sources (68.5% vs. 39.4%, *p* < 0.001) while for accuracy and completeness ChatGPT4 outperformed Claude2 (Figure 1). For group 1 questions, Claude2 had a higher rate of agreement/strong agreement compared to ChatGPT4 (73.4% vs. 63.5%, *p* < 0.001). Conversely, for group 2 questions, ChatGPT4 exhibited a higher rate of agreement/strong agreement than Claude2 (71.3% vs. 67.9%, *p* = 0.018). Notably, ChatGPT4's agreement/strong agreement rate was significantly higher for the accuracy and completeness parameters in group 2 questions, whereas it was lower for the sources parameter across both question groups.

**TABLE 2:** Percentage of raters reporting agreement/strong agreement (score 4–5), overall and according to domain.

Stakeholder	Overall	Domain					
		Accuracy	Clarity	Completeness	Relevance	Sources	Usefulness
All							
ChatGPT	67.4%	67.3%	86.4%	64.6%	78.5%	39.4%	68.1%
Claude2	70.7%	61.7%	86.9%	58.5%	79.6%	68.5%	68.8%
McNemar test	*p* = 0.002	*p* = 0.044	*p* = 0.916	*p* = 0.028	*p* = 0.696	*p* < 0.001	*p* = 0.864
Group 1							
ChatGPT	63.5%	60.8%	80.4%	62.1%	73.3%	32.5%	71.3%
Claude2	73.4%	64.2%	84.2%	62.5%	77.5%	75.0%	77.5%
McNemar test	*p* < 0.001	*p* = 0.456	*p* = 0.289	*p* = 1.000	*p* = 0.237	*p* < 0.001	*p* = 0.064
Group 2							
ChatGPT	71.3%	73.8%	92.5%	66.7%	83.8%	46.3%	65.0%
Claude2	67.9%	59.2%	89.6%	55.0%	81.7%	62.1%	60.0%
McNemar test	*p* = 0.018	*p* < 0.001	*p* = 0.296	*p* = 0.003	*p* = 0.559	*p* < 0.001	*p* = 0.219

Interrater reliability was good for most of the parameters for both chatbots, as measured by the interclass correlation coefficient (Table 3).

**TABLE 3:** Interrater reliability (intra‐class coefficients).

Parameter	Intraclass correlation coefficient (95% CI)	
	ChatGPT4	Claude2
Accuracy	0.83 (0.72–0.91)	0.86 (0.77–0.92)
Clarity	0.77 (0.64–0.88)	0.66 (0.45–0.82)
Relevance	0.72 (0.55–0.82)	0.79 (0.67–0.89)
Completeness	0.63 (0.41–0.80)	0.71 (0.54–0.84)
Sources	0.95 (0.92–0.97)	0.92 (0.88–0.96)
Utility	0.69 (0.50–0.83)	0.76 (0.61–0.87)

Regarding readability, ChatGPT4 presented lower complexity than Claude2 (FKGL mean score 4.57 vs. 6.05, *p* < 0.001) requiring an easy‐fairly easy English in 93% of cases compared to 80% for Claude2. Moreover, ChatGPT4 produced shorter sentences and longer responses compared to Claude2, while Claude2 generates more complex sentences and more concise answers (Table 4).

**TABLE 4:** Flesch Kincaid Calculator evaluation.

	ChatGPT	Claude2	*p* *
Flesch–Kincaid Grade Level			
Mean (SD)	4.57 (1.01)	6.05 (1.18)	0.003
Flesch Reading Easy score			
Mean (SD)	81.44 (5.75)	76.84 (7.28)	0.041
Average Words per Sentence			
Mean (SD)	10.74 (1.53)	14.15 (2.34)	< 0.001
Words			
Mean (SD)	306.27 (26.49)	188.20 (35.60)	< 0.001

* Paired Student's *t*‐test.

## 4 Discussion

The study undertook a comparative assessment of the information produced by ChatGPT4 and Claude2 when presented with a set of questions pertaining to reconstructive surgical procedures for cancers affecting the head and neck region. While Claude2 showed an overall superiority in providing sources, ChatGPT4 scored significantly higher in terms of accuracy and completeness for group 2 questions. Furthermore, ChatGPT4 recorded a higher percentage of agreement/strong agreement among raters for group 2 questions, despite Claude2 having a higher overall percentage of agreement, which substantially depended on its greater reliability in providing bibliographic sources. Finally, ChatGPT4's responses were found to be more readable and comprehensible, requiring an easier level of English.

Regarding group 1 queries, although statistically significant differences did not emerge for the vast majority of parameters between the two chatbots, the fact that ChatGPT4 provides information in a more easily understandable manner makes it potentially more usable for a non‐specialist population. Furthermore, the higher accuracy and completeness of ChatGPT4's responses to questions from Group 2 make it a suitable tool even for use by head and neck surgeons. One of the most significant advantages of Claude2 over ChatGPT4 is its ability to provide reliable bibliographic sources. However, when it comes to questions in Group 2, around one‐third of evaluators did not agree that this was a strength for Claude2 in this area.

A recent study thoroughly evaluated the performance of AI language models including ChatGPT and Claude AI, across four key metrics in complex medical decision‐making scenarios. The study found that both Claude AI and ChatGPT delivered excellent results, outperforming the other AI systems tested, suggesting that Claude AI and ChatGPT are currently among the top AI language models for complex medical decision‐making applications [18].

Another study evaluated chatbots' responses to cancer pain management queries. Using questions from the European Society of Medical Oncology (ESMO) guidelines, five chatbots were tested. Despite ChatGPT4 showing the highest accuracy, all chatbots were deemed inadequate in providing accurate information for cancer patients, with resources considered quite insufficient in delivering reliable guidance to both patients and their families [19].

It should be noted that, in the present study, despite over 85% of evaluators agreeing on the clarity of the provided information, about one‐third disagreed on whether both chatbots provided accurate and complete information. This observation highlights a discrepancy between the perceived clarity of information and its accuracy and completeness underscoring the importance of evaluating the quality of information through tools that examine various parameters.

One limitation of this study is the dynamic nature of AI, which evolves continuously. Furthermore, while the 30 questions examined in this research provided a solid sample, they may not encompass all the intricacies of head and neck oncologic reconstructive surgery. Additionally, focusing on such a specialised topic might have affected the chatbots' ability to locate relevant scientific evidence and articulate responses effectively. The study highlights the need for further research to better understand the strengths and weaknesses of these chatbots in different clinical contexts and to develop strategies to maximise their effectiveness and reliability.

## 5 Conclusion

In conclusion, the results suggest that neither chatbot is clearly superior overall. However, ChatGPT4 produces more accurate and complete responses for certain question types and ChatGPT4's utilisation of simpler linguistic output may render it better suited for addressing inquiries from patients. Conversely, Claude2 outperforming on citing reliable sources. However, the substantial proportion of evaluators who disagree with the information provided by chatbots, along with experiences in other domains, suggest that such chatbots need refinement and cannot be considered an alternative to information provided by medical professionals, also in the field of oncological head and neck reconstructive surgery.

## 6 Author Contributions

**Paolo Boscolo‐Rizzo:** conceptualization of the work, development of the methodology, data curation, writing the original draft, writing the final draft, final approval. **Alberto Vito Marcuzzo:** development of the methodology, data curation, data collection, writing the final draft, final approval. **Chiara Lazzarin:** development of the methodology, data curation, data collection, writing the final draft, final approval. **Fabiola Giudici:** development of the methodology, data curation, statistical analysis, writing the final draft, final approval. **Jerry Polesel:** development of the methodology, data curation, statistical analysis, writing the final draft, final approval. **Marco Stellin:** data curation, data collection, writing the final draft, final approval. **Andrea Pettorelli:** data curation, data collection, writing the final draft, final approval. Giacomo Spinato: data curation, data collection, writing the final draft, final approval. **Giancarlo Ottaviano:** data curation, data collection, writing the final draft, final approval. **Marco Ferrari:** data curation, data collection, writing the final draft, final approval. Daniele Borsetto: data curation, data collection, writing the final draft, final approval. **Simone Zucchini:** data curation, data collection, writing the final draft, final approval. **Franco Trabalzini:** data curation, data collection, writing the final draft, final approval. Egidio Sia: data curation, data collection, writing the final draft, final approval. **Nicoletta Gardenal:** data curation, data collection, writing the final draft, final approval. **Roberto Baruca:** data curation, data collection, writing the final draft, final approval. **Alfonso Fortunati:** data curation, data collection, writing the final draft, final approval. **Luigi Angelo Vaira:** development of the methodology, data curation, data collection, writing the final draft, final approval. **Giancarlo Tirelli:** development of the methodology, data curation, data collection, writing the final draft, final approval.

## Disclosure

No copyrighted works owned by third parties have been included.

## Ethics Statement

The study was performed in alignment with the Helsinki Declaration principles and did not necessitate ethics committee approval, as it involved neither patients nor animals.

## Consent

The authors have nothing to report.

## Conflicts of Interest

The authors declare no conflicts of interest.

### Peer Review

The peer review history for this article is available at https://www.webofscience.com/api/gateway/wos/peer‐review/10.1111/coa.14261.

## Supporting information


**Supporting Information 1:** List of Questions and ChatGPT4 Responses.

**Supporting Information 2:** List of Questions and Claude2 Responses.

**Supporting Information 3:** AI’s scores and *t*‐Student test calculation.

## Data Availability

The data that support the findings of this study are available from the corresponding author upon reasonable request.
