# Supplementary material for: Quality of Information Provided by Artificial Intelligence Chatbots Surrounding the Reconstructive Surgery for Head and Neck Cancer: A Comparative Analysis Between ChatGPT4 and Claude2
Source: Clin Otolaryngol. 2024 Dec 4;50(2):330–5. doi: 10.1111/coa.14261 (PMC11792429; doi:10.1111/coa.14261)
Supplement: Supplementary file 3 — Supporting Information 3: AI’s scores and t‐Student test calculation. [file COA-50-330-s001.docx]

**Supplementary Table 1**

|  | Group 1 | | | Group 2 | | |
| --- | --- | --- | --- | --- | --- | --- |
| Question | Mean score  ChatGPT4 | Mean Score Claude2 | p-value* | Mean score  ChatGPT4 | Mean Score Claude2 | p-value* |
| q1 | 25.44 | 23.50 | 0.053 | 23.57 | 23.25 | 0.847 |
| q2 | 21.06 | 23.69 | 0.016 | 22.80 | 19.10 | <0.001 |
| q3 | 17.44 | 20.69 | <0.001 | 23.10 | 21.40 | 0.087 |
| q4 | 23.06 | 21.44 | 0.072 | 23.75 | 22.69 | 0.517 |
| q5 | 20.94 | 24.56 | 0.012 | 24.38 | 23.06 | 0.050 |
| q6 | 25.63 | 23.56 | 0.011 | 21.88 | 20.94 | 0.096 |
| q7 | 21.00 | 21.94 | 0.416 | 23.50 | 22.31 | 0.224 |
| q8 | 21.56 | 24.56 | <0.001 | 22.88 | 22.94 | 0.904 |
| q9 | 21.94 | 22.63 | 0.201 | 25.15 | 23.38 | 0.002 |
| q10 | 24.56 | 25.81 | 0.036 | 23.31 | 21.44 | 0.010 |
| q11 | 21.69 | 24.56 | 0.014 | 22.06 | 21.88 | 0.628 |
| q12 | 22.94 | 24.06 | 0.034 | 23.06 | 25.00 | 0.022 |
| q13 | 22.81 | 22.44 | 0.804 | 23.13 | 24.75 | 0.060 |
| q14 | 22.94 | 25.50 | 0.009 | 24.44 | 24.88 | 0.508 |
| q15 | 22.00 | 24.63 | 0.004 | 25.13 | 24.75 | 0.713 |

*paired Student's t-test
